# Changes in quality of life and health status in patients with extracorporeal life support: A prospective longitudinal study

**DOI:** 10.1371/journal.pone.0196778

**Published:** 2018-05-10

**Authors:** Kang-Hua Chen, Yu-Ting Chen, Shu-Ling Yeh, Li-Chueh Weng, Feng-Chun Tsai

**Affiliations:** 1 School of Nursing, College of Medicine, Chang Gung University, Tao-Yuan, Taiwan; 2 Department of Cardiovascular Surgery, Chang Gung Memorial Hospital, Tao-Yuan, Taiwan; 3 Department of Psychiatry, Chang Gung Memorial Hospital, Tao-Yuan, Taiwan; 4 Department of Nursing, Chang Gung Memorial Hospital and Chang Gung University of Science and Technology, Tao-Yuan, Taiwan; 5 Department of General Surgery, Chang Gung Memorial Hospital, Tao-Yuan, Taiwan; 6 Department of Cardiovascular Surgery, Chang Gung Memorial Hospital and Chang Gung University, Tao-Yuan, Taiwan; National Yang-Ming University, TAIWAN

## Abstract

**Background:**

Extracorporeal life support (ECLS) provides emergency pulmonary and cardiac assistance for patients in respiratory or cardiac failure. Most studies evaluate the success of ECLS based on patients’ survival rate. However, the trajectory of health status and quality of life (QOL) should also be important considerations. The study’s aim was to explore changes in health status and QOL in adult patients weaned from ECLS who survived to hospital discharge over a one-year period.

**Study design:**

A prospective longitudinal study was conducted from April 2012 to September 2014. A convenience sample of patients who had undergone ECLS was followed for one-year after hospital discharge. Heath status was measured with a physical activity scale, the Centre for Epidemiologic Studies Depression scale, and a social support scale; we assessed quality of life with the physical and mental component summary scales of the Short-Form 36 Health Survey. Changes in depression, social support, physical activity and QOL were analysed with generalized estimating equations at 3-month intervals; participants’ QOL at 12 months after discharge was compared with the general population.

**Results:**

A total of 231 patients received ECLS during the study period. Sixty-five patients survived to hospital discharge (28% survival rate); 32 participants completed the study. Data showed scores for physical activity increased significantly over time (*p* < .001), while depression and social support significantly decreased (*p* < .05 and *p* < .001, respectively). Participants with veno-venous ECLS had higher scores for depression than participants with veno-arterial ECLS (*p* < .05). PCS scores significantly increased at 9, and 12 months after discharge (*p* < .05 and *p* < .001, respectively). There was no significant change in MCS scores.

**Conclusions:**

This was a preliminary study of patients with ECLS following hospital discharge over a one-year period. One year following hospital discharge survivors of ECLS continued to experience physical complications and some continued to have depressive symptoms; the level of social support was significantly lower after hospital discharge. Healthcare professionals should understand the trajectory of health status and QOL after discharge, which can help developing evidence-based interventions and improve QOL for survivors of ECLS.

## Introduction

Cardiac or respiratory disease patients in critical condition need comprehensive medical care. Extracorporeal life support (ECLS) is a cardiopulmonary bypass procedure, which can provide additional time for diagnosis and treatment of cardiac disease [1] and rescue therapy for acute respiratory or cardiac failure and extracorporeal cardiopulmonary resuscitation (ECPR) [2–3]. The rate of survival for adults from discharge or transfer can vary depending on the form of ECLS: 58% for respiratory, 41% for cardiac and 39% for ECPR [3]. In Taiwan the survival rate for adults has been reported to be 20–64% at the time of hospital discharge [4–7], and 25.5–63.6% one year after hospital discharge [8–9].

ECLS can result in hemorrhage (e.g., hemothorax, gastrointestinal or intracranial bleeding, cannula access sites), neurological complications (e.g., stroke, encephalopathy), organ failure (e.g., acute renal failure), ischemic necrosis of lower extremities, and infections [2,10–13]. In addition, a patient’s underlying illness and the course of treatment in the intensive care unit (ICU) can impact outcomes. In order to understand the impact of ECLS on patient outcomes, studies have focused on predictors of in-hospital mortality and weaning from ECLS [6,14–15], survival rate [6–8], complication rate [13,16–17], physical and psychological health status [13,16–17], and quality of life (QOL) [13,16–17].However, there have been no longitudinal studies on QOL for patients with ECLS after discharge.

Patients may have several distinct health trajectories depending on the amount of time since discharge (e.g., one month, 12 months). Survivors of ECLS are reported to have significantly lower QOL based on scores for the physical and psychological domains of the Short-Form 36 (SF-36) when compared with a healthy population [11,13,17]; however, QOL for ECLS survivors was better than for patients with other serious or chronic diseases [11,18]. One qualitative study followed adult patients who survived ECLS for one year following hospital discharge; patients experienced different forms of physical and psychological stress, which varied with time after discharge [19]: 50% experienced lower limb problems at 6 months; and a small number continued to have limb weakness and constraints in joint movement at 12 months; and one patient’s health improved by 9 months, but his psychological status was poor, he lacked friends, and was unable to work full-time. Thus, it is important to understand changes in patterns of patients’ health conditions for the past, present, and projected future [20].

Evaluating the health outcome of ECLS survivors is a complex and important process. Bandura’s social cognitive theory emphasizes the formation and modification of human behaviors in a social context. Behavioral outcomes (e.g., QOL) are guided by the interaction of three influences: personal, environmental, and behavioral [21]. Personal influences include affective factors (e.g., depression); environmental influences involve social factors (e.g., social support); and behavioral influences are the actions and reactions of an individual (e.g., physical activities). Theory-based research can provide a better understanding of interventions and their actual effects [22]. Therefore, identification of long-term changes in depression, social support, and physical activity in adult survivors of ECLS following hospital discharge could help healthcare professionals predict the impact of personal, environmental, and behavioral influences on the trajectory of survivors’ QOL.

To examine health outcomes for adult patients who had been weaned from ECLS and survived to hospital discharge this longitudinal study explored: 1) survival rate at the time of hospital discharge, 2) ICU outcomes following ECLS for survivors, and 3) changes in depression, social support, physical activity and QOL during a one-year period following hospital discharge. Accurate understanding of changes in health status is an essential requirement for maintaining and improving the quality of health services [23]. Identifying patients’ health status characteristics could be used to develop comprehensive interventions to affectively meet social and behavioral needs of survivors of ECLS at different stages following hospital discharge.

## Materials and methods

This was a longitudinal and descriptive study, which followed patients over a one-year period following hospital discharge after treatment with veno-arterial (VA) or veno-venous (VV) ECLS. Data analysis of participants and non-participant survivors was performed on data collected from April 2012 to September 2014. Data on participant survivors was collected between April to December 2012 and May 2013 to September 2014. During the period between April 2012 and April 2013 a few attending physicians were uncooperative regarding identification of patients who survived ECLS and were discharged; therefore we were unable to extend an invitation to these 15 survivors to participate in our study.

### Participants

Survivors of ECLS were recruited from thoracic and cardiovascular wards of one medical center in northern Taiwan by convenience sampling prior to hospital discharge. The attending doctors of the thoracic or cardiovascular department recommended patients to the researcher. Patients eligible for the study were ≥ 20 years of age (the legal age of adults in Taiwan is 20), received ECLS as a result of a diagnosis of acute refractory cardiogenic shock or respiratory failure; patients expressed voluntarily consent to participate, and were able to speak Mandarin or Taiwanese. Because the ability to communicate (verbally or non-verbally) and complete the questionnaires was required for data collection, any limitation in communication or cognitive impairment, either as a result of pre-existing conditions or a result of ECLS procedures, excluded survivors from participating.

### Data collection

A trained research assistant collected data from chart review and questionnaires after participants provided signed informed consent. Pre-discharge data was collected 3–7 days prior to hospital discharge. Pre-discharge baseline demographic data and clinical characteristics of participants were obtained from patients’ charts; measures of physical activity, social support and depressive symptoms were determined from patient questionnaires (described below) at a time convenient for the participant. In addition, participants’ telephone numbers were collected for post-discharge contact; arrangements were made by phone for the collection of post-discharge data at a time convenient for each participant. Post-discharge data was collected either in participants’ homes or the outpatient department at 3-month intervals (3, 6, 9, and 12 months following hospital discharge). These post-discharge surveys included the questionnaires regarding the participants’ physical activity, status of depression, social support, and QOL.

#### QOL questionnaire

Participants’ QOL was measured using scales for the physical and mental component summary (PCS and MCS, respectively) of the Short-Form 36 (SF-36) Health Survey questionnaire [24]. The PCS scale consists of four health concepts: physical functioning (PF), role disability due to physical health problems (RP), bodily pain (BP) and general health (GH). The MCS scale includes four health concepts: vitality (VT), social functioning (SF), role disability due to emotional problems (RE) and mental health (MH). PCS and MCS scores were calculated using norm-based scoring methods with Taiwan-specific SF-36 algorithms [25]. Scores in each subscale range from 0 to 100, with higher scores representing better health outcomes [25]. The SF-36 survey collects data regarding a person’s QOL for the past month. To eliminate the variable of length of hospitalization, the initial data collection for participants’ QOL was 3 months following hospital discharge. Additional measures were collected at 6, 9, and 12 months.

#### Measures of depressive symptoms, social support, and physical activity

Depressive symptoms were measured using the Centre for Epidemiologic Studies Depression scale (CES-D) [26]. The CES-D is a 20-item measure that asks participants to rate how often they experienced symptoms associated with depression, such as restless sleep, poor appetite, and feeling lonely over the past week. Each question has a set of four answers that cover a range of severity of depression. The total score ranges from 0 to 60, with a higher score representing more severe depressive symptoms. The CES-D also provides cut-off scores (e.g., 16 or greater) that aid in identifying individuals at risk for clinical depression, with good sensitivity and specificity and high internal consistency [27].

A 14-item Social Support scale was used to determine perception of effective social support received by participants. The scale measures four types of functional assistance: emotional (4 items), appraisal (3 items), informational (4 items) and tangible (3 items). Scores for each item range from 0 to 4; higher scores indicate a greater perception of effective social support [28].

Participants’ physical activity was measured using a two-part Functional Status Questionnaire: part one measures basic activities of daily living (BADL, 3 items) and part two measures intermediate activities of daily living (IADL, 6 items). Scores for each item range from 0 to 4. Scale scores for each part, along with the single-item scores, are displayed in a summary score; higher scores represent greater functional ability [29].

### Statistical analysis

Statistical analyses were conducted using IBM SPSS, version 22.0, for analysis (IBM SPSS Statistics for Windows, Version 22.0. Armonk, NY: IBM Corp). Means, standard deviations (SD), standard error (SE) and percentages were used for descriptive statistics of participants’ characteristics, ICU outcomes associated with ECLS, status of depression, social support and physical activity. Mann-Whitney U tests or chi-square tests were used to examine the homogeneity between participants and non-participant survivors of ECLS. Comparison of participants’ health status and quality of life and different socio-demographic characteristics and ECLS variables was performed using Mann-Whitney U tests or Pearson correlation test. One-sample t-test was used to compare the differences in QOL for the subscales of the SF-36 between the reference value of the general population in Taiwan [25] and study participants at the four time points following hospital discharge. Finally, we used generalized estimating equations (GEE) to analyze the changes of depression, social support, physical activity and QOL over time among adult patients who had been weaned from ECLS during a one-year period following hospital discharge.

### Ethics approval and consent to participate

The Institutional Review Board (IRB) of Chang Gung Memorial Hospital approved the study before the data collection was undertaken (Approval number 100-3191B). A researcher explained the purpose of the study and informed patients that they could withdraw from the study at any time without any reason or suffering any consequences in care. Patients were assured their data would be kept confidential. After patients received this information and agreed to participate they signed a written informed consent.

## Results

**Fig 1:**
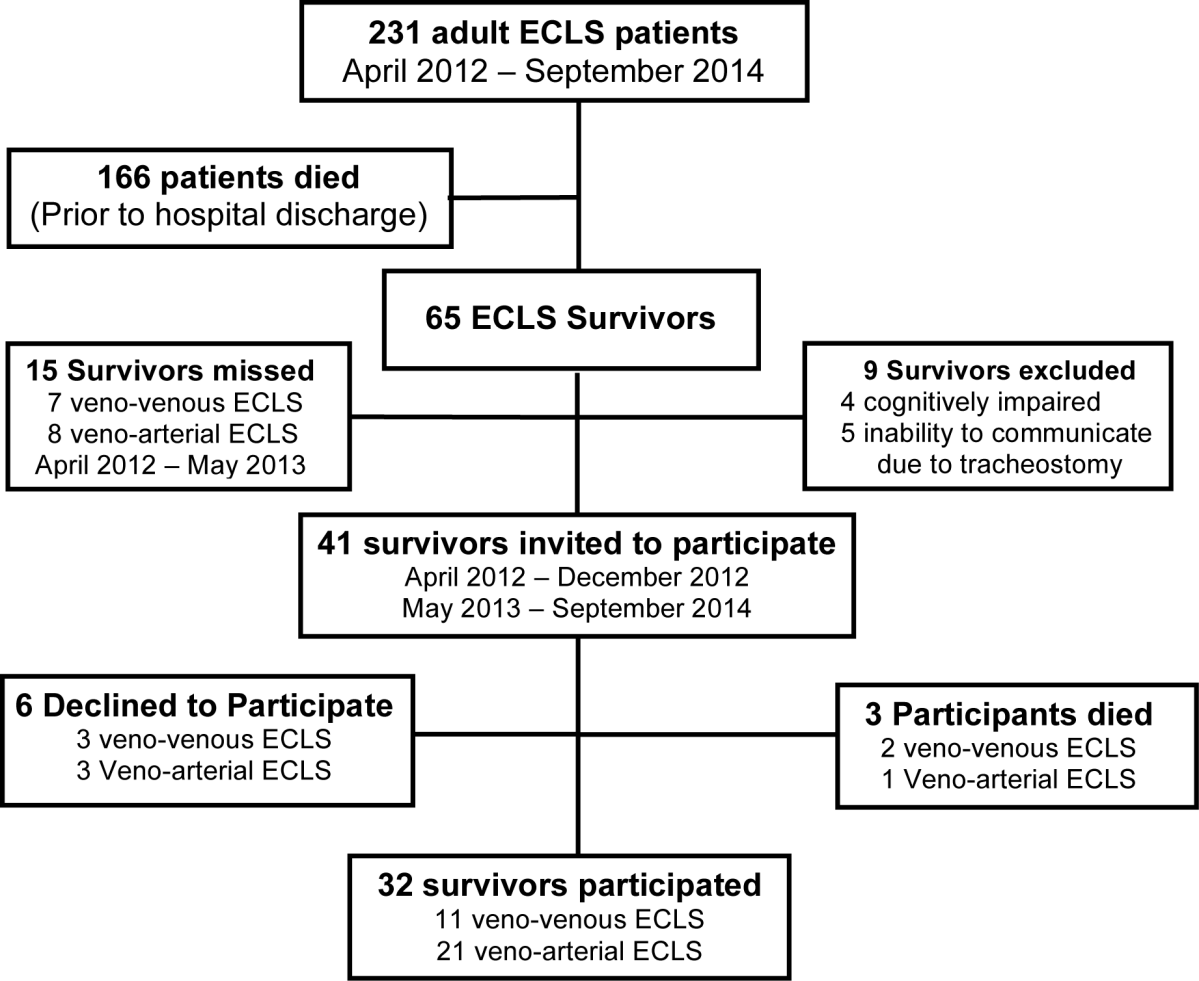
Flowchart of patients receiving ECLS during the study period. Survivors (65) included participants who completed the study (32), participants who died before completion of the study (3) and excluded patients (9). Non-participants (21) included missed patients (15) and those who declined to participate (6).

From April 2012 to September 2014, 231 adult patients received ECLS; 166 patients died before hospital discharge and 65 ECLS patients survived to hospital discharge; the survival rate was 28%. Of the 65 survivors, 15 were not identified at the time of the study and 5 survivors with tracheostomies were too weak to communicate either verbally or non-verbally. A total of 32 survivors participated in the study; 41 survivors met the inclusion criteria, however 6 declined to participate and 3 died before completion of the study. The flowchart describing the outcomes for the ECLS patients, survivors, and participants is shown in Fig 1.

Table 1 shows the age, gender, and clinical outcomes for all survivors of ECLS during the study period (n = 65) as well as comparisons of participants (n = 32) and non-participants (n = 21). Most participants were male (84%), which was significantly greater (*p* < .05) than for non-participants (57%, n = 12); gender was the only significant difference between the two groups. The mean length of ICU stay for participants was 17.4 days; mean duration of hospitalization was 48.1 days. An intra-aortic balloon pump (IABP) was used for 44% of participants before VA ECLS to improve cardiac function. A total of 56% of participants received distal perfusion and there were no leg amputations. ECLS requires support with endotracheal intubation and mechanical ventilation; the mean duration was 15.7 days.

**Table 1:** Age, gender, and clinical variables for survivors of ECLS discharged from hospital, and participants compared with non-participants.

Variable	Survivors^a^ (n = 65)	Excluded/Died^b^ (n = 12)	Participants (n = 32)	Non-participants^c^ (n = 21)	*p-*value^d^
Age (years), mean ± SD	54 ± 14	62 ± 10	52 ± 12	51 ± 17	.740
Males, n (%)	50 (77)	11 (92)	27 (84)	12 (57)	.028*
BMI, mean ± SD	26.3 ± 5.4	24.9 ± 3.1	27.2 ± 6.6	25.6 ± 4.2	.363
IABP prior to VA ECLS, n (%)	29 (46)	5 (33)	14 (44)	8 (38)	.543
Distal perfusion, n (%)^a^	36 (57)	6 (50)	18 (56)	12 (57)	.838
SOFA score	10.1 ± 3.2	11.7 ± 3.7	9.8 ± 2.8	9.6 ± 3.2	.619
LVEF prior to discharge (%), mean ± SD	53.2 ± 15.5	51.7 ± 12.8	52.5 ± 18.5	51.0 ± 17.0	.475
Duration of intubation (days), mean ± SD	19.0 ± 18.0	35.5 ± 29.5	15.7 ± 13.3	14.2 ± 8.2	.654
Duration of ECLS (days), mean ± SD	7.3 ± 5.2	7.4 ± 6.3	8.0 ± 5.9	6.0 ± 2.8	.154
Duration of ICU stay (days), mean ± SD	22.0 ± 16.5	38.6 ± 27.3	17.4 ± 9.7	19.5 ± 10.3	.459
Duration of in-hospital stay (days), mean ± SD	52.7 ± 42.0	88.3 ± 53.6	48.1 ± 41.7	39.0 ± 19.7	.370
Reason for ECLS support, n (%)					.371
Cardiac shock before ECLS	35 (54)	5 (42)	19 (60)	11 (52)	
Respiratory failure before ECLS	25 (38)	4 (33)	11 (34)	10 (48)	
ECPR	5 (8)	3 (25)	2 (6)	0 (0)	

BMI, body mass index; ECLS, extracorporeal life support; ECPR, extracorporeal cardiopulmonary resuscitation; VA, veno-arterial; IABP, intra-aortic balloon pump; LVEF, left ventricular ejection fraction; SD, standard deviation; SOFA, sepsis-related organ failure assessment.

^a^All patients who received ECLS between April 2012 and September 2014 and were discharged from hospital.

^b^Survivors who received ECLS but did not meet the study criteria (9) and died after discharge (3)

^c^Survivors who declined to participate (6), or were not available to be invited to participate (15)

^d^The p value was determined by Mann-Whitney U tests or chi-square test for comparisons of age, sex and ICU outcomes between participants and non-participants.

*p < .05

Baseline demographics, ECLS variables, and differences in health status and quality of life for participants are shown in Table 2. The mean age of participants was 52 years (SD = 12). Approximately one-third of participants (37%) had two or more comorbidities; these included diabetes mellitus (38%), hypertension (38%), end-stage renal disease (6%), or other diseases (6%) such myocardial infarction. Most participants (66%) had been treated with VA ECLS. Socio-demographic characteristics of education and marital status were significantly related to social support pre-discharge (*p* < .05 and *p* < .01, respectively). When we examined the variables of ECLS, participants with VV ECLS had higher scores for depression than participants receiving VA ECLS (*p* < .05).

**Table 2:** Participants’ (n = 32) demographics and ECLS variables at baseline and compared with health status (pre-discharge) and quality of life (3 months post-discharge).

			Health Status Variables, Pre-discharge									Quality of Life Variables (3 months post-discharge)					
			Physical Activity			Social Support			Depressive Symptoms			PCS			MCS		
Variables	Mean ± SD	n(%)	M	SE	*p-*value^a^	M	SE	*p-*value^a^	M	SE	*p-*value^a^	M	SE	*p-*value^a^	M	SE	*p-*value^a^
Baseline demographics																	
Gender					.960			.920			.801			.614			.880
Male		27(84)															
Female		5(16)															
Age, years	52 ± 12				.406			.654			.536			.664			.232
Education (n/%)					.208			.031*			.208			.081			.611
High School and below		21(66)															
College and above		11(34)															
Marital status					.742			.006**			.386			.321			.805
Married		23(72)															
Other^b^		9(28)															
Living with family					.688			.875			.563			.375			.375
Yes		31(97)															
No (n/%)		1(3)															
Employed					.472			.650			.570			.677			1.00
Yes (n/%)		19(59)															
No (n/%)		13(41)															
ECLS variables																	
Type of ECLS					.254			.434			.016*			.815			.667
Veno-venous		11(34)	18.1	2.0		54.0	3.6		19.5	3.6		44.4	2.1		56.2	1.8	
Veno-arterial		21(66)	21.0	1.5		57.1	1.6		10.3	1.6		42.3	2.0		53.9	1.6	
Comorbidities (3 months)					.477			.224			.774			.954			1.00
≦1 (n/%)		20(63)															
≧2 (n/%)		12(37)															
Cardiac or Pulmonary rehabilitation					.785			.088			.434			.938			.254
Yes (n/%)		9(28)															
No (n/%)		23(72)															

SD, standard deviation; MCS, mental component summary scales of the Short-Form 36; PCS, physical component summary scales of the Short-Form 36; SE, standard error; ECLS, extracorporeal life support.

^a^ p-value calculated with Mann-Whitney U test for comparison of two variables, or Pearson correlation test for continuous variables

^b^Divorced, separated, single.

*p < .05

**p < .01

**Fig 2:**
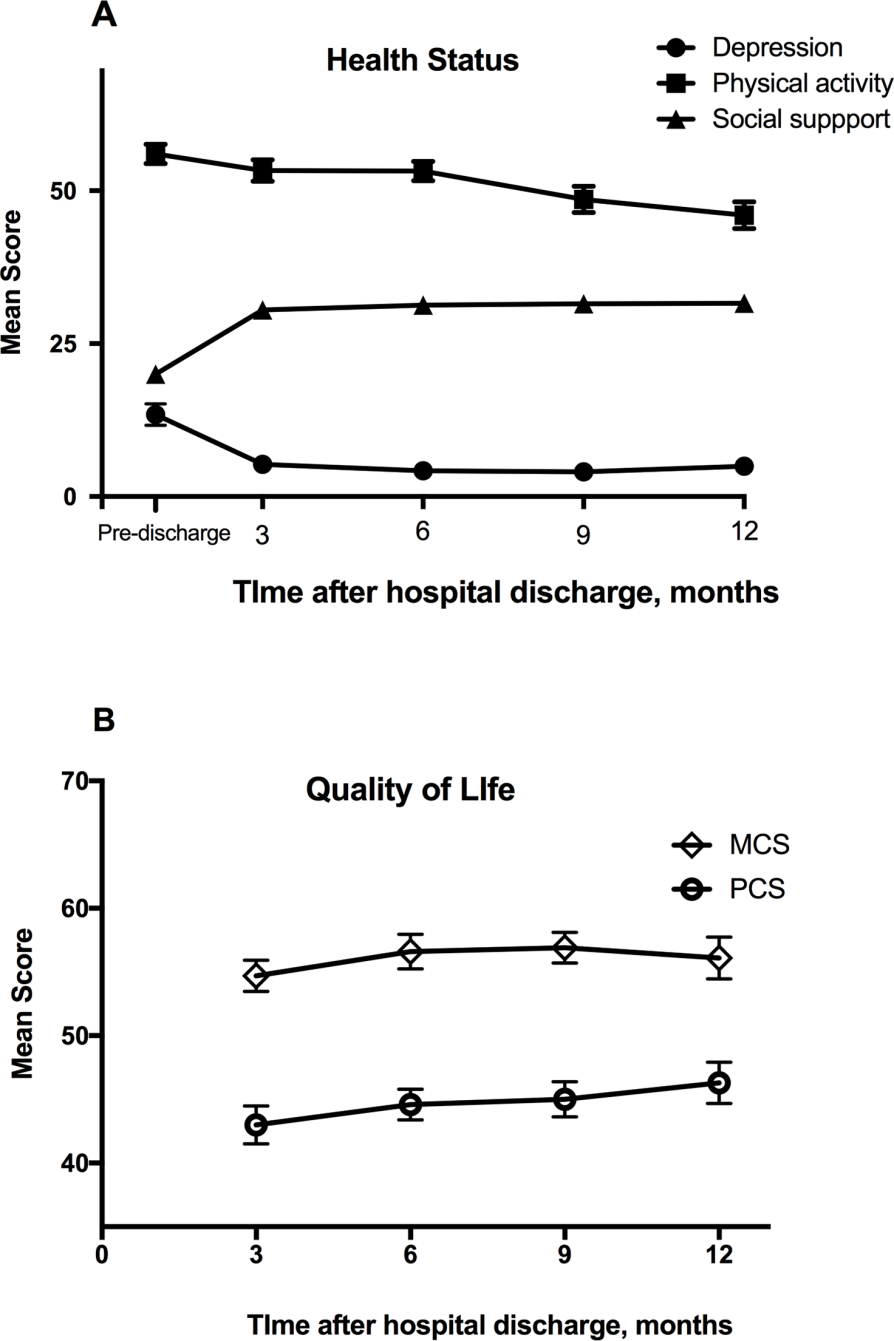
Health outcomes for long-term survivors of extracorporeal life support over one year following hospital discharge. Mean scores for measures of health status (**A**) and quality of life (**B**). PCS, physical component summary; MCS, mental component summary.

Table 3 shows scores for participants’ health status pre-discharge and QOL at 3, 6, 9, and 12 months post-discharge. Prior to discharge, the mean total score for physical activity was 20.0 (55.6% of the total score; SD = 6.9), indicating greater effort was required to recover BADL and IADL. Mean scores increased significantly over time (*p* < .001) and the trajectory of the level of physical activity level was consistently higher than pre-discharge levels at all times post-discharge (Table 3, Fig 2). The mean total score for depression pre-discharge was 13.4 (SD = 9.9). Nine participants (28%) reported a high level of depressive symptoms (CES-D score ≥ 16). Scores for CES-D significantly decreased over time (*p* < .001). When compared with pre-discharge scores, CES-D scores significantly decreased at 3, 6, 9, and 12 months after discharge. The mean total score for social support pre-discharge was 56.0 (80% of total score; SD = 9.0). The level of social support was significantly lower between pre-discharge scores and 9 months after discharge (*p* < .01) and between pre-discharge scores and 12 months after discharge (*p* < .001); a significant change over time was also observed (*p* < .001). Quality of life was significantly higher as measured by PCS scores at 9 months (*p* < .01) and 12 months (*p* < .001); there was no significant difference between scores for the MCS.

**Table 3:** Generalized estimating equation analysis of changes in mean value for health status and quality of life of participants following ECLS.

		Time after discharge			
Variable	Pre-discharge	3 months	6 months	9 months	12 months
Health Status					
Physical activity (mean ± SD)	20.0 ± 6.9	30.5 ± 7.2***	31.3 ± 7.0***	31.5 ± 7.4***	31.6 ± 7.2***
Depression (mean ± SD)	13.4 ± 9.9	5.3 ± 5.5***	4.2 ± 4.0***	4.0 ± 5.0***	5.0 ± 6.8***
Social support (mean ± SD)	56.0 ± 9.0	53.3 ± 9.9	53.2 ± 9.0	48.6 ± 12.1**	46.0 ± 12.1***
Quality of life					
PCS (mean ± SD)		43.02 ± 8.5	44.6 ± 6.9	45.0 ± 7.8**	46.3 ± 9.0***
MCS (mean ± SD)		54.70 ± 6.9	56.6 ± 7.0	56.9 ± 6.8	56.1 ± 9.1

PCS: physical component summary scales; MCS: mental component summary scales; SD: standard deviation.

**p < .01

***p < .001

GEE analysis was performed to examine the longitudinal effects of ECLS on health status and quality of life following hospital discharge (Table 4). Physical activity significantly increased from pre-discharge over time and at 3, 6, 9, and 12 months post-discharge (*p* < .001); social support was significant from pre-discharge over time (*p* < .001) and post-discharge at 9 months (*p* = .004) and 12 months (*p* < .001). Because baseline scores for depressive symptoms were higher for participants treated with VV ECLS than VA ECLS (Table 2), analysis of changes in depressive symptoms over time included a subgroup analysis using group (VA vs. VV), time, and group × time as explanatory variables in the GEE model to examine the difference in depressive symptoms between participants treated with VA ECLS and VV ECLS (Table 4). The results showed that group had a significant effect on depression (*p* < .05). Scores for CES-D significantly decreased over time (*p* < .001). Depression exhibited a significant group × time effect (*p* < .01), especially from pre-discharge to 3 months (*p* < .001), 6 months (*p* < .01), and 12 months after discharge (*p* < .05).

**Table 4:** General estimating equation model for analyzing the effect of ECLS on variables of health status and quality of life.

Variable	β	SE	95% CI		*p*
Health Status					
Physical activity^a^					
3 month vs. pre-discharge	10.53	1.31	7.96	13.1	< .001
6 month vs. pre-discharge	11.28	1.34	8.66	13.9	< .001
9 month vs. pre-discharge	11.47	1.29	8.95	13.99	< .001
12 month vs. pre-discharge	11.76	1.32	9.17	14.35	< .001
Depression^b^					
Group (VA vs. VV)	-9.17	2.34	-13.75	-4.59	< .001
Time					
3 month vs. pre-discharge	-15.36	2.22	-19.72	-11.01	< .001
6 month vs. pre-discharge	-15.36	2.54	-20.35	-10.38	< .001
9 month vs. pre-discharge	-12.91	2.64	-18.08	-7.74	< .001
12 month vs. pre-discharge	-12.82	2.67	-18.04	-7.59	< .001
Interaction					
VA × 3 month	10.98	2.74	5.61	16.35	< .001
VA × 6 month	9.36	3.14	3.21	15.52	.003
VA × 9 month	5.34	3.26	-1.04	11.72	.101
VA × 12 month	6.54	3.30	0.06	13.02	.048
Social support^c^					
3 month vs. pre-discharge	-2.72	2.07	-6.78	1.34	.189
6 month vs. pre-discharge	-2.84	2.34	-7.44	1.75	.225
9 month vs. pre-discharge	-7.41	2.57	-12.44	-2.37	.004
12 month vs. pre-discharge	-10.33	2.24	-14.72	-5.95	< .001
Quality of life					
PCS^d^					
6 month vs. 3 month	1.60	1.03	-0.421	3.62	.121
9 month vs. 3 month	1.93	0.94	0.10	3.76	.039
12 month vs. 3 month	3.37	0.26	2.85	3.88	< .001
MCS^e^					
6 month vs. 3 month	1.88	1.51	-1.09	4.84	.215
9 month vs. 3 month	2.20	1.55	-0.83	5.24	.155
12 month vs. 3 month	1.44	1.51	-1.51	4.39	.338

ECLS, extracorporeal life support; SE, Standard error; VA, veno-arterial; VV, veno-venous

MCS, mental component summary scales of the Short-Form 36; PCS, physical component summary scales of the Short-Form 36.

^a^Model effect of VA group × time interaction: Wald chi-squared = 108.90, *p* < .001

^b^Model effect of VA group × time interaction: Wald chi-squared = 17.50, *p* = .002

^c^Model effect of VA group × time interaction: Wald chi-squared = 90.12, *p* < .001

^d^Model effect of VA group × time interaction: Wald chi-squared = 253.66, *p* < .001

^e^Model effect of VA group × time interaction: Wald chi-squared = 2.71, *p* = .439

Longitudinal effects of ECLS on quality of life were examined by comparing scores at 3 months after hospital discharge with scores at 6, 9, and 12 months. Quality of life as measured by PCS scores increased over time (*p* < .001) and the increase was significant at 9 and 12 months after discharge, compared with 3 months. Scores for the MCS improved between 3 months and 6, 9, and 12 months after discharge (β = 1.88 at 6 months; β = 2.20 at 9 months; β = 1.44 at 12 months). However, the effect of time was not significant.

**Fig 3:**
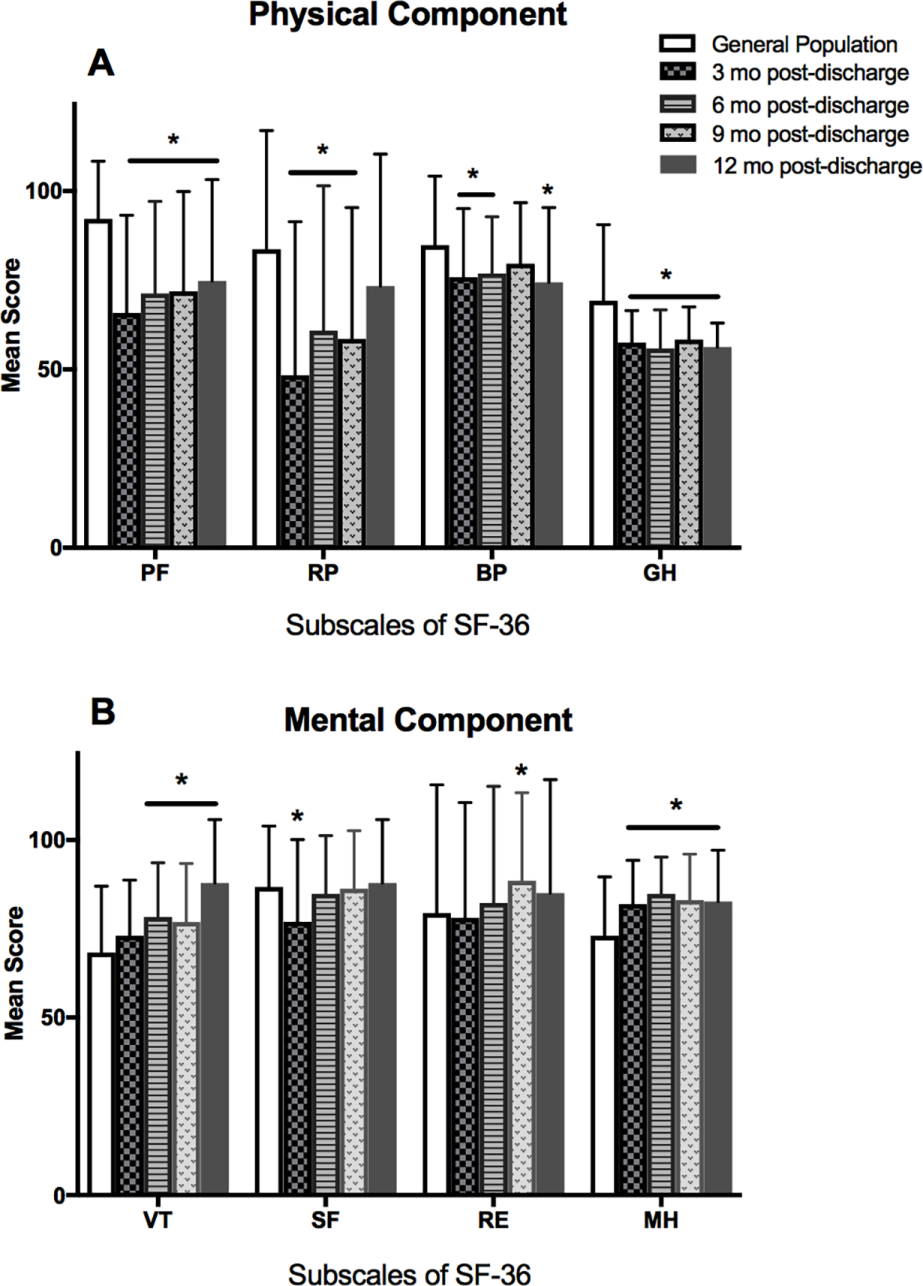
SF-36 subscale scores for quality of life over time. Subscale scores for Physical (**A**) and Mental components (**B**) of the SF-36 for participant survivors of extracorporeal life support at 3, 6, 9 and 12 months compared with scores for the general population of Taiwan [30]. PF: physical functioning; RP: role disability due to physical health problems; BP: bodily pain; GH: general health; VT: vitality; SF: social functioning; RE: role disability due to emotional problems; MH: mental health; *p < .05.

Participant survivors’ subscale scores for the physical and mental components of the SF-36 at 3, 6, 9 and 12 months were compared with the general population in Taiwan [25] (Fig 3). Six months after discharge, participants’ QOL was poorer than the general population for all four physical components. And by 12 months after discharge participants had significantly lower scores for three of the components (physical functioning, bodily pain, and general health). The mental component score for mental health was significantly higher at all time points after discharge; vitality was higher at 6, 9 and 12 months.

## Discussion

### Survival rate at hospital discharge

This was a small, single-center long-term follow-up study of adult patients who received VA or VV ECLS. The survival rate for patients receiving ECLS and being discharged from the hospital was 28%, which is similar to an earlier study conducted in Taiwan [4], but lower than the Extracorporeal Life Support Registry Report for 2016 and a report from the Bureau of National Health Insurance of Taiwan in 2011 [3,31]. Patients who underwent ECLS in Taiwan during 2004–2006 accounted for over 50% of the total number recorded around the world during that time period [32]. ECLS is considered standard treatment in Taiwan because national health insurance provides reimbursement, however it is important for healthcare professionals to define what indications should be required for using ECLS as an intervention, without regard to family or social pressure [31].

### Changes in health status and quality of life

We compared the health status and QOL for survivors of ECLS with socio-demographic characteristics after hospital discharge. Social support was significantly related to level of education and marital status. College educated and above participants had higher levels of social support than high school and lower participants. Higher education has been shown to be associated with higher socio-economic status and social capital, which often coincides with greater availability of social support [33]. The relationship between marital status and social support might be explained by the fact that married persons in Taiwan live with extended families who provide support; family and healthcare professionals have been shown to be the most important resources of social support for patients [34].

Over the 12 months following discharge, depressive symptoms declined and physical activity continued to improve. Our findings are consistent with a previous study [19], which demonstrated by one year following discharge, most ECLS survivors were able to take control of their lives and adjust to a new lifestyle. It is important to note that the mean total score for depression at 12-months was similar to the score at 3-months after discharge. Four participants (12.9%) reported a high level of depressive symptoms (CES-D ≥ 16). The prevalence of depression was lower than past studies [16–17], however symptoms of depression have been shown to be significantly related to long-term cardiac mortality [35].

Survivors’ QOL, measured by PCS and MCS scores, were higher one year following hospital discharge than reported for ECLS patients in previous studies [11,23]. However, the mean MCS score was less than 60% of the total score and the mean PCS score was less than 50% of the total score. PCS scores increased significantly at 9 and 12 months after discharge. Adult ECLS patients have been reported to experience physical and psychological stress, especially during the first 6 months following hospital discharge, and at 12 months a small number continue to have complications of lower limb weakness and constraints in joint movement [19], which might lead to a reduction in overall general health for more than 3 years after hospital discharge [11]. PCS scores might also result from worry about experiencing another incidence of ECLS, which requires self-motivation for successful rehabilitation and emotional adaptation for their mental health after discharge [19].

Although MCS scores improved at 6, 9, and 12 months after discharge compared with 3 months, the effect of time was not significant. The physiological discomforts of ECLS can lead to psychological stress and social isolation requiring an extended time to cope with these stresses [19]. Providing holistic medical assessment and management for survivors of ECLS could assist with recovery and provide information about self-care and ECLS-treatment-related resources (e.g. rehabilitation centers, support groups, complementary, and alternative medicine) before discharge [13,19]. These strategies could decrease the length of time of a patient’s discomfort from complications as well as provide broader support.

Three months following discharge, five of the eight subscale scores of the SF-36 were lower for ECLS survivors than the general population of Taiwan suggesting a poorer QOL; however scores for mental health were significantly higher. Twelve months after discharge three of the subscale scores for physical health remained significantly lower; vitality and mental health was higher. This suggests that survivors of ECLS exhibit good mental health one year following discharge, however physical QOL is still compromised.

Participants received ECLS as a result of different conditions (e.g., acute respiratory or cardiac failure), and therefore the impact on health outcomes may differ. Our data showed that depressive symptoms were significantly lower in participants treated with VA ECLS than participants with VV ECLS and scores for both groups were significantly different over time. Past studies have shown depression is prevalent in 4–28% of patients with VV ECLS [17, 36–37] and 11–27% of patients with VA ECLS [16,30–38]. However, none of these studies compared the prevalence and severity of depression between VA and VV ECLS. Bandura’s social cognitive theory emphasizes human behavior is influenced by reciprocal determinism, and is determined by the interaction of depression, physical activity and social support [21]. Because the level of social support was significantly lower at 9-months and 12-months after discharge, it is important for healthcare professionals to continually monitor depressive symptoms in ECLS survivors.

### Study limitations

Our results have some limitations. The sample size was relatively small; however, GEE analysis is an efficient statistical method for determining significant changes in variables for small samples [39]. Because this is the first quantitative and preliminary longitudinal study of patients with ECLS following hospital discharge over a one-year period, we felt analysis of a small sample was warranted. Another limitation results from the lack of cooperation from some of the attending cardiovascular physicians from April 2012 to April 2013, which prevented us from having the opportunity to invite 15 patients to participate in our study. In addition, several patients were not surveyed due conditions of cognitive impairment before ECLS (n = 4), the presence of a tracheostomy (n = 5), which resulted in their inability to communicate by verbal or nonverbal methods; and six refused to participate; this may have resulted in an overestimation of results for QOL and health status [11]. The internal validity of the results may be limited by the number of patients who survived to hospital discharge, but declined to participate (n = 6), or were not given the opportunity to be invited to participate (15). Comparisons of participants and non-participants showed that, with the exception of the number of males, there was no difference in demographics; and there was no difference in ICU outcomes associated with ECLS between the two groups (Table 1). Although 84% of participants were male, no gender differences have been found in other studies of depression and QOL in ECLS patients [12–13,16–17].

## Conclusions

Physical activity in survivors of ECLS improved during the 12 months after discharge and depressive symptoms significantly declined over time and exhibited a significant interaction between groups (VA vs. VV) and time. In addition, QOL significantly increased, which was demonstrated by the increase in PCS scores after 9 and 12 months. However, there was no improvement in MCS scores. Our findings support Bandura’s social cognitive theory regarding interactions between health outcomes and QOL. Early identification of patient’s needs may promote QOL and most participants (75%) experienced physical complications during the first 3 months after hospital discharge. We suggest healthcare professionals take an active role in the holistic assessment of patients’ physical-psychological-social needs, evaluate the underlying conditions of VA and VV ECLS, and arrange for appropriate cardiac or pulmonary rehabilitation for long-term follow-up as soon as possible after transfer from the ICU, which could have a positive impact on a patient’s health status and QOL [13,19]. Structured exercise rehabilitation programs and healthcare professionals’ support can aid patients recovering from critical illness and avoid distress from the physical complications resulting from ECLS [13,19].

## Supporting information

S1 File (SAV)
